# Geometrical model establishment and preoperative evaluation on A-T flap design: Finite element method-based computer-aided simulation on surgical operation processes

**DOI:** 10.3389/fsurg.2022.988783

**Published:** 2022-10-12

**Authors:** Yan Zhao, Zonglin Yang, Lifen Chen, Yuhui Peng

**Affiliations:** ^1^ Department of Dermatology Surgery, The First Affiliated Hospital of Fujian Medical University, Fuzhou, China; ^2^ School of Mechanical Engineering and Automation, Fuzhou University, Fuzhou, China

**Keywords:** A-T flap, geometrical model, mesh, finite element, skin tension, deformation, stress

## Abstract

**Objective:**

A-T flap has been extensively applied to repair dermal soft tissue defects. The flap design completely depends on the experience of doctors. Herein, we explored the approach of analyzing the reasonability of A-T flap design and performed a simulation of operation processes by computer-aided technology. Afterward, the finite element analysis software (MSC.Marc/Mentat) was used to establish the simulation model, based on which the computer simulation of flap suturing and release state in A-T flap surgery was performed.

**Methods:**

A geometrical model of the A-T flap was established, and the length-width ratio of the flap, maximum suture distance, and suture area that could influence the postoperative suture effects of the flap were analyzed. The reasonable surgical planning for A-T flap design based on the crossing constraint relationship was achieved. The simulation model was established by the finite element analysis software (MSC.Marc/Mentat), based on which computer simulation of flap suture and release state of A-T flap in surgery processes were performed. The flap’s stress and deformation distribution results confirmed the applicability of the A-T flap design method proposed in the present study.

**Results:**

When the apex angle of the A-T flap was 60 degrees, the suture area was the smallest, and the flap design had the highest practicability.

**Conclusion:**

Computer-assisted preoperative assessment, which has high clinical value, could provide a theoretical basis for A-T flap design in clinical practice.

## Introduction

Skin acts as a barrier that protects the body from the exterior environment and has various important functions such as regulating body temperature and sensing external stimuli. Skin injuries must be rapidly repaired. As skin flaps could be used for repairing and reconstructing the injured skin, various types of skin flaps have been extensively applied in skin or plastic surgeries (1–3).

A-T flap is a local flap widely applied in clinical practice, especially for repairing dermal soft tissue injuries of the head and facial area (4, 5). However, at the moment, the design of the A-T flap and prediction of surgical outcomes, which are sometimes inappropriate and imprecise, completely depend on doctors' experience. Other factors such as the irregular morphology of the recipient surface and flap retraction could further increase the complexity of flap design. The inappropriate design can lead to adverse outcomes such as flap necrosis. Therefore, designing a flap solely based on experience has always been a challenge for doctors. For a long time, researchers have been waiting to develop an effective method that could predict the postoperative effects of A-T flap and precisely guide the design of A-T flap.

Regarding the application of finite element analysis in surgery, Keeve (6) and Koch (7) made great efforts to simulate the deformation process of skin and soft tissue in mandible plastic surgery based on finite element method. A simplified finite element model of the head and face was constructed to explore the application of the finite element method in simulating plastic surgery (8). Rubén (9) analyze and compare the different distribution of von Mises stress and displacement of the cartilage in healthy and damaged (with Cam-type) human hip joints by use the FEM on basis of MSC/Marc software. Fátima (10) proposed a parameterized three-dimensional FE model to analyze the influence of sex, age, weight and height on the medium-sized human lumbar functional spinal unit behavior. William (11) use FEM to analyze the difference in stiffness and behavior under loading between a lateral vs. ventral plate fixation, with unlocked screws and different gap scenarios for double pelvic osteotomy.

In the aspect of flap design, Tepole et al*.* (12, 13) have established the finite element models of direct advancement flap and the double back-cut flap. He investigated the stress distribution during the advancement of these two types of flaps, revealing that for the direct advancement flap, the maximum stresses occurred at the distal end of the flap, while for the double back-cut flap, the maximum stresses occurred at the lateral edges of the flap. Yet, they did not extract the geometrical model of the two flaps to guide the proper flap design. In their study, Pauchot et al*.* (14, 15) proposed a simple numerical model for the V-Y flap, suggesting the apex of the flap as the essential factor for the V-Y flap design. Finite element simulation showed that the 18.8 kPa vertical iso-stress line was directly associated with the regions of tissue necrosis. Yang et al*.* (16) established a geometrical parameter model of V-Y flap for the investigation of V-Y flap design, and the crossing constraint relationship of geometrical parameters showed that the optimal strategy of flap apex was 60° for V-Y flap design. In addition, the finite element analysis results also validated the practicability of the V-Y flap design method. The local advancement of the A-T flap refers to applying external forces on dermal soft tissues to induce mechanical creep and elastic stretching, thus inducing substantial deformation and displacement. However, previous studies failed to effectively investigate the reasonability of A-T flap design and preoperative assessment.

The aim of this study was to summarize the experience related to the length-width ratio of the flap, maximum suture distance, and suture area of A-T flap in clinical practice through the geometrical parameter model of A-T flap, and decide the A-T flap design strategy at the ideal skin elasticity through the crossing constraint relationship. We also performed simulation analysis of the stress on the flap, suture, and release state of the A-T flap in operation processes using the finite element analysis software, which could promote postoperative effects and validate the A-T flap design method.

## Materials and methods

### Materials

**Figure 1:**
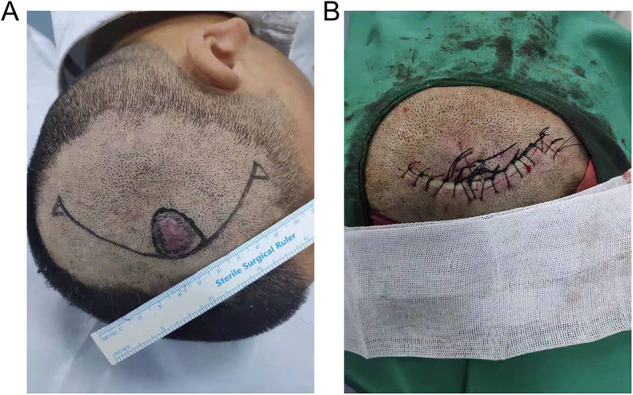
A-T advance flap operation. (**A**) Image before operation. (**B**) Image after the operation. A copy of the written consent is available for review by the Editor-in-Chief of this journal.

**Figure 2:**
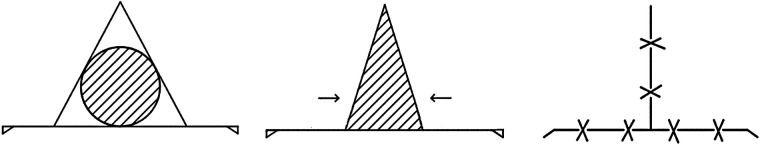
Operation process.

In the present study, we focused on the geometrical model of the A-T flap. The typical A-T flap application is shown in Figure 1A, where the round black area represents the skin tumor, and the red line the preoperative design of the surgical route, which had an “A” shape. The shape of a scar after suture showed a “T” shape (Figure 1B). The operation processes for the A-T flap mainly consisted of 3 following steps (Figure 2): first, the wound was trimmed to a triangle, the incision was elongated to bilateral sides along the bottom edge, and the length of the incision was generally about 3 folds of the diameter of the defect (5). Second, the bilateral waists of the triangle were pulled inward, and finally, all the edges were sutured.

**Figure 3:**
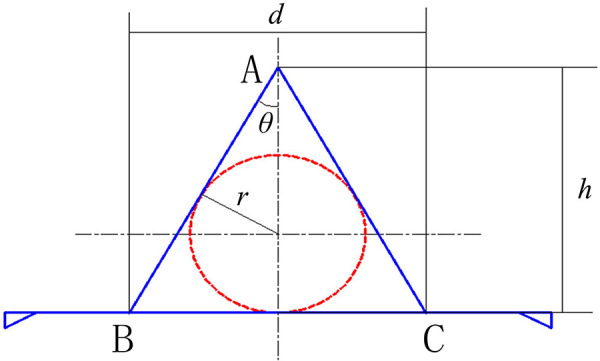
Geometrical model of the A-T flap.

The corresponding geometrical model of A-T flap is shown in Figure 3. The round area formed by the red dash line shows the site of the skin tumor, and the triangle area in the middle that was formed by the blue line shows the corresponding defect. To prevent the “cat ear” shaped folds at the bilateral ends of the line after suture, two small incisions of right triangles were kept on bilateral sides. The area of “cat ear” was generally very small and did not require resection (17). Therefore, the influences of “cat ear” on the model could be neglected. In Figure 3, *h* indicates the length of the flap, and *d* indicates the width of the flap (BC).

### Methods

#### Key geometrical parameters of A-T flap

(1) *Length-width ratio of flap*: the length-width ratio of the flap (*h/d*) is generally an important factor in flap surgeries. The *h/d* determines the shape and size of the flap and influences the reasonability of the flap design. According to the geometric model, the relationship between *h/d* and apex angle of the flap is as follow:

(1) $$\frac{h}{d}=\frac{1}{2\tan\theta }\;$$

(2) *Maximum suture distance*: the width of the flap (*d*) represents the maximum suture distance, which is decided by the radius of the incision of the skin tumor (*r*) and the degree of apex angle of the flap (2*θ*). The maximum suture distance is also an important factor influencing the postoperative recovery effects. The following equation was obtained from the equation (1) and geometrical relationship in Figure 3:

(2) $$d\;=2r\left(\frac{1+\sin\theta }{\cos\theta }\right)\;$$

(3) *Suture area*: the area of triangle ABC in Figure 3 is the suture area (*S*). The suture area has a relatively substantial influence on the postoperative recovery of the incision. As shown in the geometrical model, the bilateral waists of the triangle are tangent to the skin tumor, and the suture area (*S*) is as follow:

(3) $$S\;=\;r^{2}\frac{{(1+\sin\theta )}^{2}}{\sin\theta \cdot \cos\theta }\;$$

#### Design of A-T flap

The relationships of the 3 geometrical parameters (*h/d*, *d*, and *S*) with *θ* showed that the strategy of A-T flap design was directly related to the radius of skin tumor incision and apex angle of the flap. Assuming that the radius of incision of skin tumor (*r*) was 1 cm, different values of length-width ratio (*h*/*d*), maximum suture distance (*d*) and suture area (*S*) and the apex angle were shown in Table 1. Obviously, apex angle of 60° meted with the requirement of smallest suture area. At the same time, the strategy of A-T flap design, i.e., the apex angle of flap should meet various constraint relation of clinical experience (please refer to the Discussion section). In such a situation, the apex angle of the A-T flap was 60°, making the triangle an equilateral triangle, and the width of the flap (*d*) was 3.46 cm, and the length of the flap (*h*) was 3 cm.

**Table 1:** Comparison of geometrical parameters of A-T flap.

Length-width ratio -h/d	Maximum suture distance -d (mm)	Suture area -S (mm^2^)	Apex angle-2*θ* (°)
1.37	28.56	5.60	40
1.07	31.39	5.28	50
0.87	34.64	5.20	60
0.71	38.42	5.27	70
0.60	42.89	5.48	80

### Finite element analysis of A-T flap

The local advancement of the A-T flap applies external forces on dermal soft tissues to induce the mechanical creep and elastic stretching, inducing substantial deformation and substantial displacement and therefore allowing the suture of the incision. To better assess the preoperative A-T flap design strategy introduced in the present study, the finite element analysis software was used to establish the finite element model of A-T flap advancement, based on which the computer simulation of the suture and release state of A-T flap during operation processes were performed to investigate the stress and deformation distribution of flap, thus investigating the practicability of the A-T flap design strategy.

#### Finite element model of A-T flap

**Figure 4:**
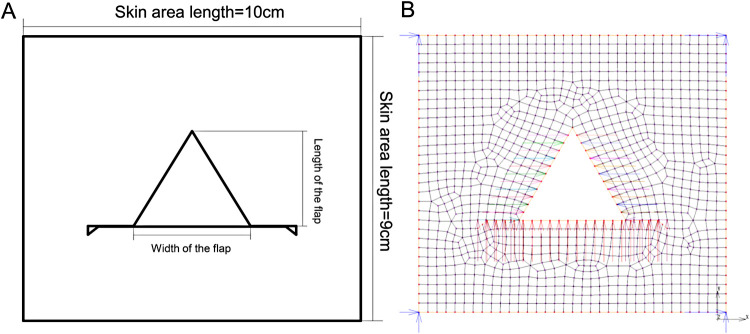
A-T flap whole model. (**A**) Schematic diagram of A-T flap. (**B**) Diagram of finite element meshing. Length of skin area; length of the flap; width of the flap; length of skin area.

As the skin thickness is far smaller than the skin size, the skin model could be considered as an approximate two-dimensional planar model. The height and width of the flap were 3 and 3.46 cm, respectively, and the apex angle of the flap was 60°. As the area distal from suturing was under relatively low stress and deformation, the influence on postoperative effects was relatively low, and only the area of 3-fold of flap size was investigated. A rectangular model of the skin of the surgical area with the size of 9 cm × 10 cm was established by the finite element analysis software (MSC.Marc/Mentat). As to the element type during meshing, it is generally considered that 4-node quadrilateral elements hold good calculation accuracy and efficiency, so quadrilateral element is adopted in this study. Additionally, considering that smaller elements are required to adapt to large changes in shape during the large deformation analysis, balance between the computation cost (number of elements) and the analysis accuracy should be taken into account in necessary. Therefore, four meshing schemes with side length of 4, 3, 2 and 1 mm have been carried on respectively and the sensitivity of nodes stress to element size at the release state was investigated. The results were displayed in Table 2. According to the clinical experience, the suture-bite-stitch-interval (SBSI) was about 2–5 mm (18), thus the element size of 3 mm is selected in determination, and 1,161 nodes and 1,068 quadrilateral elements were generated by meshing (Figure 4B).

**Table 2:** Von Mises and mesh quality of A-T flap FE models with different element sizes.

Element size (mm)	Elements	Nodes	Maximum von mises at release state (MPa)	Mesh quality		
				Number of upside-down elements	Number of distorted elements (Threshold = 0.5)	Number of bad aspect elements (aspect ratio >1.5)
4	564	632	2.502	0	33	2
3	1,068	1,161	1.961	0	90	0
2	2,272	2,409	2.072	0	113	0
1	4,053	4,232	2.152	0	437	0

#### Boundary conditions

During the suturing of margins of flap and incision, the mutual pulling could lead to certain displacement and deformation of flap and skin at the incision. To accurately simulate the advancement of the flap, only the four corner nodes of the flap were fixed and the concerned nodes on the incision margin were set with different values of displacement. the corresponding deformation which was caused by the movement of the above nodes enabled to completely repair the vacant area of the defect. Herein, no force was applied to the mesh model, and the definition of boundary conditions was shown in Figure 4B. According to the real operation processes of flap advancement and the analysis function of contact relationship provided by MSC.Marc/Mentat, two different node displacement constraint states corresponding to the two analysis conditions were set as follows: (1) suture: after the flap was advanced to a certain site, the additional load was applied on the incision margin and flap before suturing to induce a certain stretch and deformation of incision margin and flap. According to the corresponding positions of the incision skin edge and the flap, constraints with different displacement were applied on the nodes of margins of both incision and flap to make the nodes undergo displacements to contact to each other, and thus simulate the suture of flap and incision margin; (2) release: after the suturing was completed, the external stretch load was removed, i.e., the displacement constrictions of each node were removed, while the incision margin and flap were still adjacent to each other because of the suture. Due to the viscoelastic of the skin, the incision margin and flap were influenced by the inherent contraction effects. The displacement constrictions on all suture nodes were removed to simulate the state of the flap after external loads were released.

#### Material property

Since the compressibility of skin tissue is usually very small, and it is assumed that the mechanical properties are isotropic, the consideration of skin tissue as a kind of hyperelastic model of rubber materials is generally adopted (19). Through theoretical investigation and elaborately experimental verification, a variety of constitutive models for simulating rubber materials have been suggested, including Mooney-Rivilin model and Ogden model, but the former is suitable for small deformation situation. In addition, the formulation of Ogden model is more concise than Mooney-Rivilin model, and a large number of experiments on uniaxial tension, isometric biaxial tension and pure shear verified that Ogden model can better simulate small or considerable large deformation, which is closest to the mechanical characteristics of flap advancement (20). Therefore, the Ogden model was selected as the material model in this study. The strain-energy function of the Ogden model was as follow:

$$W=\overset{N}{\underset{n=1}{\sum }}\frac{\mu_{n}}{\alpha_{n}}[J^{\left(-\alpha_{n}/3\right)}\left(\lambda_{1}^{\alpha_{n}}+\lambda_{2}^{\alpha_{n}}+\lambda_{3}^{\alpha_{n}}\right)]-3+4.5K{\left(J^{\left(1/3\right)}-1\right)}^{2}$$

where $\alpha_{n}$ denote the exponential parameters; $\mu_{n}$ are the shear parameters;, *K* indicates the initial bulk modulus, and *J* indicates the volumetric ratio and equals to $\lambda_{1}\lambda_{2}\lambda_{3}$, and $\lambda_{i}$ (*i *= 1,2,3) mean the principal stretch ratios; *N* is the number of the terms.

For the incompressible material, J equals to 1 and the strain energy function also can be transformed as follows,

$$W=\sum_{n=1}^{N}\frac{\mu_{n}}{\alpha_{n}}[(\lambda_{1}^{\alpha_{n}}+\lambda_{2}^{\alpha_{n}}+\lambda_{3}^{\alpha_{n}})]-3$$

where *J* = 1, *N* = 2, $\mu_{1}$ = 7.5809 × 10^−7^ MPa, $\mu_{2}$ = 0.1683 MP; $\alpha_{1}$ = 2.1065, $\alpha_{2}$ = 12.006.

**Figure 5:**
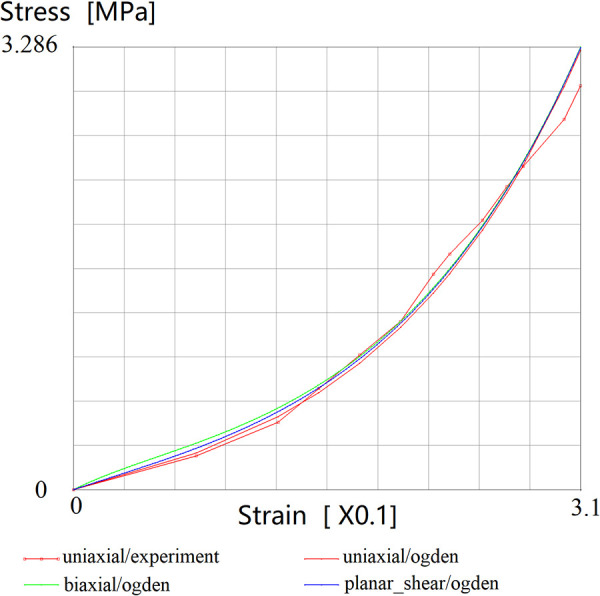
Strain-Stress fitting curves for Ogden model.

In order to simplify the calculation, the skin is assumed to be an isotropic material in this paper. The stress-strain curve of the facial skin is obtained through the uniaxial tensile biomechanical experiments (21). Consequently, the skin material parameters ($\mu_{1}$, $\mu_{2}$, $\alpha_{1}$ and $\alpha_{2}$) of Ogden model are obtained by using the material parameter curve fitting function provided by Marc program. The curve fitting results are shown in Figure 5.

## Results and discussion

### Results of computer simulation

The option “Equivalent Von Mises Stress” was selected in the analysis results, after which the results of stress and displacement were shown by the color cloud pictures.

### Suture state

**Figure 6:**
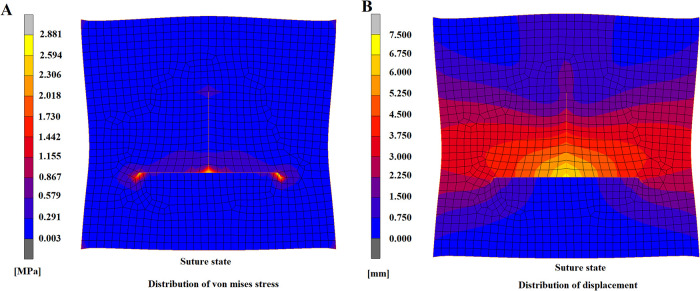
A-T flap at suturing state. (**A**) Distribution of von mises stress. (**B**) Distribution of displacement.

Regarding the overall distribution of Von Mises stress on flap model, the stress concentration points were mainly at the 4 angular points, i.e., apex of defect, central joint of suture, and suture sites of bilateral “cat ears”, which were relatively in agreement with conditions in clinical practices (Figure 6A). The displacement of the overall flap model showed symmetric displacement, and a T-shape was formed after suture, which was in agreement with the findings in clinical practice (Figure 6B). The color of cloud pictures represents the size of node displacements of the flap model, which clearly demonstrates that the bilateral skin of the defect needed to be pulled toward the center during the operation, and thus the displacements of these two parts were the highest. This was also in agreement with the previous findings in clinical practice.

### Release state

**Figure 7:**
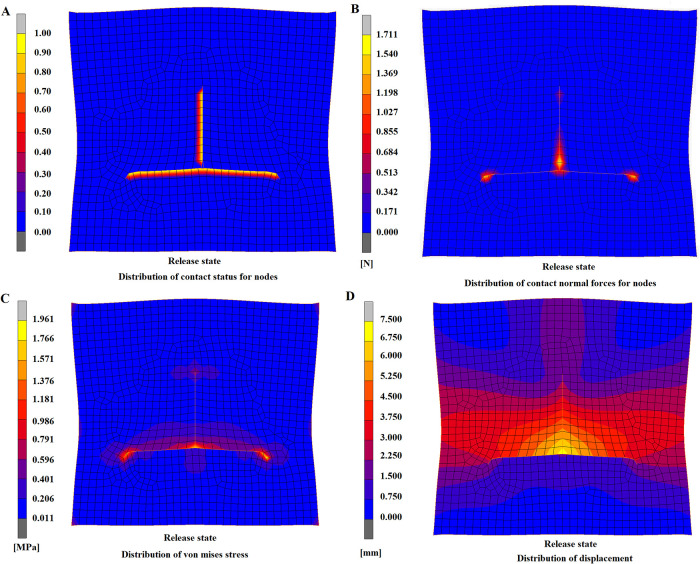
A-T flap at release state. (**A**) Distribution of contact state for nodes. (**B**) Distribution of contact forces for nodes. (**C**) Distribution of von mises stress. (**D**) Distribution of displacement.

After the constraints on displacement were removed, the T-shaped suture still maintained the even contact, and no fissure or collapse deformation was found (Figure 7A), indicating that the model had a relatively high suture effect and that this flap design strategy was practical. After the constrictions were released, skin elasticity could lead to inherent contraction effects and consequently to tension and strain. The tension mainly focused on the central suture site (Figure 7B), which not only had the maximum suture distance but was the joint of the suture of the surrounding flap. Therefore, this site was essential in influencing the recovery effects and should be considered the key suturing site. Regarding the distribution of Von Mises stress after the release of constraints, the overall trend was similar to the stress distribution in the flap model during suturing (Figure 7C); however, the maximum stress was lower than in the suture state, and the maximum von mises stress was reduced from 2.881 to 1.961 MPa. The distribution of flap displacement in release state (Figure 7D) was generally similar to the distribution during suturing (Figure 6B). By comparison of displacement distribution in state of suturing and releasing, the displacement of nodes in the lower area of bottom edge and the upper area of the apex appeared a prominent increase as the same axis scale was set for both figures. The maximum displacement after constraint release was 6.297 mm, which was 0.763 mm lower than the maximum displacement of 7.06 in the suture state. These findings indicated that after the release of constraints, certain contractions of nodes of the flap model occurred, which was consistent with the previous findings in clinical practice.

### Discussion

**Figure 8:**
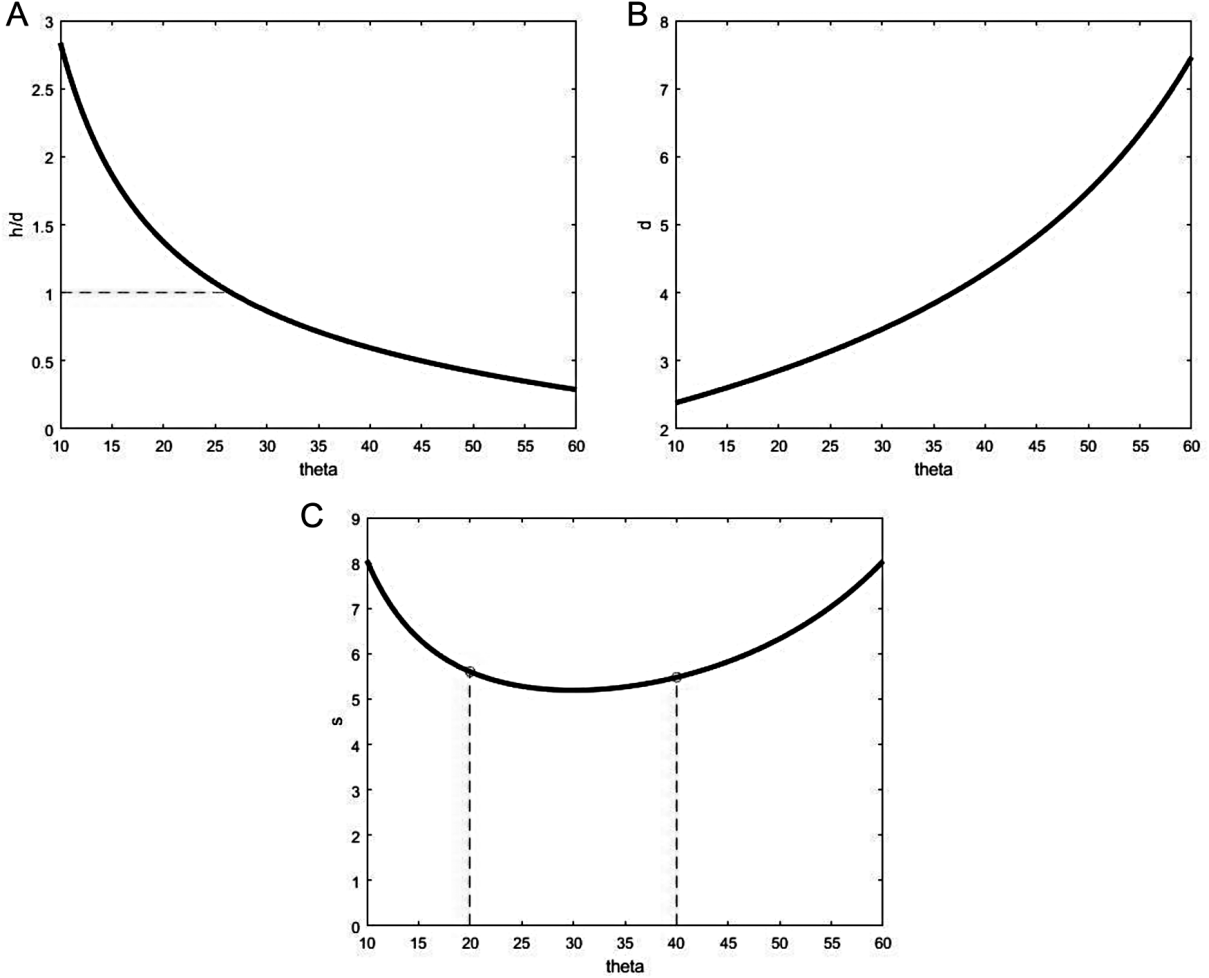
Relationship of A-T flap geometry and half apex angle. (**A**) Ratio h/d vs. ∠*θ*. (**B**) Maximum suture distance d vs. ∠*θ*. (**C**) Area S vs. ∠*θ*.

Regarding the apex angle of the flap, previous studies (15) have already shown that once the size of the incision is decided, the apex angle and length of the flap are the most important parameters for the geometrical shape of the flap. For the A-T flap design, the angle of best apex angle was 60°, where various constraint relationships were as follows: (1) the length-width ratio of the flap (*h/d*) gradually decreased with the increase of apex angle of the flap (Figure 8A). The *h/d* was generally lower than 3:1 to avoid the influence on blood supply for flap by swelling or contraction. The classic *h/d* of advancement flap was generally 1:1 or 2:1 (22). Therefore, the apex angle of the flap, i.e., should not be higher than 60°; (2) the maximum suture distance *d* gradually increased with the increase of the apex angle of the flap (Figure 8B). The lower maximum suture distance is associated with the lower stretching stress on the flap, favoring postoperative recovery. Therefore, maximum suture distance should be set at a relatively low level; (3) the suture area *S* was gradually reduced with the increase of apex angle of flap initially, after which it increased with the increase of 2*θ* (Figure 8C). The suture area should be relatively low within a proper range of *θ* (20°–40°). The lower suture area could favor the union of the wound and help reduce the scar. When the apex angle of the flap 2*θ* was 60°, the suture area of the flap was the lowest.

The biomechanical properties of skin material are the basis of finite element analysis. Aisling et al (23). tested the material mechanics of human skin samples in 56 subjects and found that the average ultimate tensile strength was 21.6 ± 8.4 MPa and average elasticity modulus was 83.3 ± 34.9 MPa. The mechanical properties of skin stretching deformation at different sites of the human back in different directions were also obtained. Furthermore, Jacquet et al*.* (24) investigated the biomechanical properties of skin in a cohort of 20 healthy volunteers with uniaxial extensometer at different anatomical sites and directions and obtained the stress-strain curves at different conditions, thus providing valuable parameters for clinical practice. Karimi and colleagues (25) calculated the viscoelastic mechanical properties of the skin tissue at the back and abdomen of rats under uniaxial loading. The distribution of elasticity modulus of the human head and facial skin is generally within the range of 3.91–13.67 kPa (26). Considering the handling of boundary conditions, the bilateral suture nodes of the flap are affected by the displacement constraints and thus symmetric, so there was no relative displacement of the flap and overall skin that influenced the deformation of the model. Therefore, no constriction was applied on the skin margin, which allowed the skin to remain at a free state that was more similar to the normal conditions. This also allowed us to evaluate the stretching and deformation of the overall skin. In the constraint release state, the constraints on the displacement of suture nodes were removed to simulate the state in which all external loads on suture nodes were removed. Our findings showed that the flap was well connected to the incision margins, and the contact was even and with no deformity. Also, the maximum stretching force on the flap was focused at the suture sites at the bottom and apex of the flap, which was consistent with the findings in clinical practice. It is worth noting that preserving some small blood vessels and nerves in suturing moderate or small area (diameter of resection area <1.5 cm) flaps could maintain the normal blood supply for flap when the flap tension was at an appropriate level. For large area flaps, the necrosis at flap margin was not associated with the flap tension but was closely associated with the diameter and pressure of perforator vessels in a flap, as well as the subdermal vascular network (27).

There are several limitations in the present study. The variations in age, gender, and skin sites in different individuals could lead to variations in biomechanical properties of skin materials, and the material properties in finite element model establishment should be set according to the individualized conditions. In addition, the A-T flap design strategy was only based on the currently available clinical experience, suture distance, and suture area, while the influence of blood vessel distribution at the operation site and texture features of skin on flap design was not considered. Moreover, for large area flaps curved incision length may be existed for A-T flap, and the tension of incision base at the “release state” may vary in different way compared to a straight base.

## Conclusions

In summary, we established the computer-assisted technology-based geometrical model of the A-T flap to investigate the three key geometrical relationships, i.e., the length-width ratio of the flap, maximum suture distance, and suture area that could influence the flap structure. The practical strategy for designing an A-T flap was obtained based on the crossing constraint relationship in clinical experience. The non-linear based finite element software was used to establish the simulation model of A-T flap, based on which the simulation analysis of flap in the suture and release states was performed, and the practicability of A-T flap design was validated by simulating the operation processes of A-T flap advancement, thus achieving the preoperative assessment of A-T flap. The findings of this study provide a new idea for designing a practical strategy for A-T flap advancement.

## Data Availability

The original contributions presented in the study are included in the article/Supplementary Material, further inquiries can be directed to the corresponding author/s.
